# The Dawn of Modern Dental Literature

**Published:** 1898-06

**Authors:** William H. Trueman

**Affiliations:** Philadelphia


### International Dental Journal.

VOL. XIX. JUNE, 1898. No. 6.

### Original Communications.[1]

### THE DAWN OF MODERN DENTAL LITERATURE.[2]

BY WILLIAM H. TRUEMAN, D.D.S., PHILADELPHIA.

ANCIENT, old, modern, and recent are convenient relative terms having, however, no absolutely fixed values. I propose to embrace by the expression Modern Dental Literature that which is in direct touch with the present; so closely in touch, indeed, that we may from the present trace backward, step by step, until a point is reached when the well-marked path abruptly ends. Beyond this sharply defined point, while we know that much concerning the teeth had been written, it is for the most part so included with other matters, so scattered and indefinite, and withal so shrouded in uncertainty, and so inaccessible, that it is difficult to trace it, and much more difficult to estimate its true value. Prior to the time when the art of printing came into general use the methods in vogue for preserving and diffusing the world's accumulated knowledge was so laborious and expensive that only matters of great moment were recorded, matters of lesser import were transmitted and preserved by word of mouth, and necessarily, on this

[1] The editor and publishers are not responsible for the views of authors of papers published in this department, nor for any claim to novelty, or otherwise, that may be made by them. No papers will be received for this department that have appeared in any other journal published in the country.

[2] Read before the C. N. Peirce Dental Society of the Pennsylvania College of Dental Surgery.

account, real progress was slow because much that was learned was forgotten. Each new generation, instead of beginning, as is now the case, its life-work where the last ended, was compelled to follow very closely the footprints of its immediate predecessors; learning by experience much of that which we now more quickly learn from the printed page, on which each generation erases the errors of the past and adds new facts for the information of its successors.

The advent of the printing-press marks an important epoch in the world's history, a momentous one in the history of its arts and its sciences. This invention, indeed, so completely revolutionized all that concerns the moral and intellectual welfare of the race, and all that makes man to differ from the brute, that we may rightly consider it the dividing line, and call all that preceded it ancient; all that followed it modern.

At this time it is very difficult to realize how the world then was, how its business was conducted, and especially the condition of its arts and its sciences, the growth and maintenance of which depends so much upon a constant interchange of thought and experience, when the only way in which this could be accomplished, other than by word of mouth, was by the slow, tedious, laborious, and expensive process of making each record, and each duplicate copy thereof, by the pen or its equivalent. Naturally, as the result, progress was slow and uncertain. The friction of mind upon mind, the constant and steady increment of knowledge, the gathering together, the comparison, the sifting of ideas and of widely separated and varied experiences, which in later times has done so much to advance the world's intelligence, was almost wholly unknown. The meager records of each succeeding dominant nationality, when its dynasty lost power and its empire crumbled, were buried beneath its ruined cities, and thus lost to their successors. Each new civilization began anew, profiting but little from its predecessors, and in turn leaving but little accessible to those which followed. Thus, civilization followed civilization in an unvaried circle, each leaving the world but little better than it found it.

Notwithstanding this, as time passed, these forward and backward movements resulted in a slow, but nevertheless a real and well-marked advance. Instruction was largely through the living teacher, and was mainly a recital of his own experience, or an exposition of the dogmas of his acknowledged master. Now and again, fostered by an enlightened monarch, brought about by commercial conditions, or under the influence of a master mind or a more than usual attractive teacher, educational centres were formed, where libraries were collected, and where, under favorable auspices, students and teachers were brought together; and here the lines were broadened, and for the time being science made a healthy and vigorous growth. For the most part, however, so far as medical science and the sciences correlated to it were concerned, dogmatic assertion and a slavish deference to authorities perpetuated error and discouraged original investigation.

As we approach the renaissance—the dawn of the nineteenth century civilization—we notice a wonderful change. The individuality of the man begins to assert itself as never before. Political and religious bonds are becoming irksome; and as the long dark night is drawing to a close, the various influences which have so long hampered intellectual progress give way before the advancing light. The political atmosphere becomes clearer, discordant elements harmonize, the world is getting together for a forward leap. The disciples of Hippocrates, Galen, and of other masters a thousand years dead are beginning to ask whether these teachers, so long implicitly followed, knew all there was to be learned. They began to ask this question of nature, to compare the accepted dogmas of the past with the experiences of the present; they began to inquire, the one of the other, Are these teachings we have so long followed really true? The freer intercourse between nations as commerce began to develop, the greater facilities for the interchange of thoughts and ideas brought about by the adoption of a universal language for science, and the ready duplication of records afforded by the printing-press soon brought about such an upheaval and revolution as this world of ours had never before known.

Of the extent and character of the dental literature of ancient times but little is now known. It is said of many medical writers of antiquity that they wrote upon the teeth. It is not probable, however, that any of these books have been preserved in their entirety; indeed, very little of their writings of any kind or character have survived, and this little has necessarily passed through so many revisions and expositions that it is difficult, if not impossible, to give an intelligent idea of what they originally were. Of many of their books not a trace can be found; all we know is the title or, perhaps, a few thoughts or suggestions preserved through quotations of later writers. Even of the great teachers, all that has come down to us are the more prominent of their discoveries, or the aphorisms, or distinctive doctrines taught as theirs by their professed disciples. Traces of a dental literature may be found in these, and here and there an intimation that the teeth and their diseases had occupied their thoughts. Now and again an historic incident, a joke or pun of some ancient jester, or an antique tomb, gives evidence that our art existed and was practised in remote times; to what extent, the character of the work and all details concerning it have been so far completely lost. We may hope that this chapter of dental history will be brought to light as the records of long-forgotten civilizations are discovered and deciphered.

We have in this little book I now show you, the oldest printed book known to dental bibliography, dated 1532, evidences of this pre-existent dental literature of which I have spoken. It is in German; translated, the title-page reads, "Teeth Medicine; against all kinds of defects and diseases of the teeth; many wholesome and well tried medicines extracted out of the books of Galen, Avicenna, Mesue, Cornelius Celsus, Pliny, and others; together with compendious and useful instructions as to how one can keep his teeth healthy, and how one can extract the bad hollow teeth which get to paining easily."

This copy was printed by Peter Jordan, at Meyng. I have seen another copy, dated 1541, printed by Christopher Egenolff, Frankfurt; and I find record of another publication under date of 1614, slightly differing in title, but undoubtedly the same. We thus see that it was kept before the public some eighty years at least. For a few minutes let us examine this curious and quaintly worded little book; let us see who these worthies were to whom it refers, and what they are credited with saying. First, however, permit me to say that it was written for lay readers, by an anonymous writer, and belongs to a class of publications upon medicine and allied subjects widely differing in value; while the best of them have their use as educators of the public, they have no standing in professional literature. This belongs to the better class, and historically, apart from being an excellent specimen of early printing, introduces us to the teachers and teachings of dental science as presented to laymen three centuries and a half ago.

Claudius Galen was in continental Europe for more than a thousand years a recognized authority in medical science. He is supposed to have died, in his seventy-first year, about A.D. 200. He was an extensive writer and an original investigator. He did much to systematize the study of anatomy, urging the importance of dissecting the cadaver, and insisting that the bones could be studied only from the skeleton. It is said that he wrote a better account of the teeth than any which had hitherto existed. He taught that they were formed during the period of gestation; but that they remained hidden in the aveoli until after birth; that the molars of the upper jaw had three roots, those of the lower only two; and that the canine teeth had also received the name of ocular or eye-teeth, because branches are sent to them of a nerve which distributes others to the eye. He has a long chapter on their forms and their evolution, and does not hesitate to pronounce them true bones.[1]

Avicenna, the Latinized form of his Arabic name, Eben Sina, was born in Bokharra in 980, and had a checkered career; at one time loaded with favors, and again cast into prison and threatened with execution.

He was, however, a remarkable man and a brilliant scholar. His mental activity took in a wide range. He was master of all that pertained to the classics and sciences of his age and race. As a medical writer his reputation rests chiefly upon his "System of Medicine," a voluminous work, embracing anatomy, physiology, materia medica, and an exposition of all the diseases to which man is subject. Originally written in Arabic, it was later translated into Hebrew and Latin, and for six centuries continued to be the recognized text-book of medical science through Asia and Saracenic Europe. No author since Galen had enjoyed so wide or so extensive an authority in the medical world. Like many another genius, while expounding the laws of health to others and teaching them how to live, he sadly neglected to practise his own precepts. After spending the day in close study and research, his nights were devoted to dissipation and pleasure. Although possessing a sound and vigorous constitution, this disregard of his own teachings ended his career at the early age of fifty-seven.

Regarding Mesue there is much confusion. There were two of that name, both Arabian physicians, and both writers upon medical topics. The first was born in 777, and died about 857; the other Mesue lived about a century later. It is very difficult, if possible, with any degree of accuracy to separate the writings of these two distinguished men. We have not in full the writings of either, and that which we have, through its many rewritings and translations, has been so thoroughly intermingled and undergone so many verbal changes that it fails to correspond with quotations of contemporaneous writers which otherwise would serve to establish their identity. The earlier Mesue wrote more extendedly and confined himself more closely to medicine.

---

[1] Nasmyth on the Teeth. London, 1839, p. 3.

Cornelius Celsus is supposed to have lived during the first half of the first century of the Christian era, the exact date is not known. "Roman literature, otherwise so barren of good medical authorities, can boast of possessing in Celsus one who for elegance, terseness, learning, good sense, and practical information stands unrivalled." The above quotation, from an able writer, indicates his place in medical literature. He wrote a large work, somewhat on the plan of an encyclopædia; of this, unfortunately, the medical part alone has come down to us, in eight volumes, comprising a history of the various medical schools which had arisen before his time, rules for preserving the health, and the general symptoms and phenomena of diseases, diet, surgery, etc.

Johannes de Figo, a name not found on the title-page but freely quoted in the text, is the Germanized name of Giovanni da Vigo,[1] an eminent surgeon, a native of Genoa, who, in 1503, was invited to Rome by Pope Julius the Second, who made him his first surgeon. His work, entitled "Practica in Arte Chirurgica copiosa," first published at Rome (1514) in folio, became extremely popular and was very frequently reprinted, both in the original and in translation. While making these notes I had before me a number of editions ranging in date from one in old Latin, dated 1521, entitled "Opera domini Joanis de Vigo in Chyrurgia," to a translation in English, dated London, 1571. It contains a short chapter on the anatomy of the teeth and jaws, and a much more extended one on toothache; from this the quotations in the little book are taken. In this chapter, cleaning cavities of decay and filling them with gold leaf to preserve the remaining portion of the tooth is referred to in much the same terms as the quotation credited to Mesue. It is in such dissertations as this that the world's accumulated dental knowledge, prior to the advent of a distinctive dental literature, has been transmitted to us. They are well worth a careful study.

Pliny was not a medical writer. He was born A.D. 23, and although all his life in the employ of the state was a prolific writer. Among his many works was one upon natural history, published A.D. 77. It is from this work the extracts in this little book have been taken. Its contents were of very unequal value, details that are strange and wonderful rather than those really important having often attracted the author's attention.

Whoever the author of this old dental book was, he has con-

---

[1] Such lingual variations in proper names are quite common, and are at times embarrassing. This work, in some editions, is known as "Vigon's Surgery."

fined himself in making his extracts to strictly standard writers, and in so doing simply followed the custom of medical writers of his day. It was not a progressive age, but rather one in which age and antiquity commanded more respect than real merit. We will now rapidly glance over its contents.

It is divided into thirteen chapters, with titles as follows:

I.—When the Teeth grow, and how many a Man has.
II.—The Various Causes of Bad Teeth.
III.—How to assist Children that their Teeth may grow easily.
IV.—Of Toothache.
V.—Of Hollow Teeth.
VI.—Of Teeth on Edge.
VII.—Of Yellow and Black Teeth.
VIII.—Of so-called Sleeping Teeth.
IX.—Of Loose Teeth.
X.—For Worms in the Teeth.
XI.—Ulceration, Bad Smell, and Decay of the Gums.
XII.—How to draw Bad Teeth.
XIII.—How to save the Teeth.

The preface reads, translated, as follows: "The God that is almighty, the God to whom all things from the beginning are known, has been pleased in his wisdom to so order that the lower animals and likewise men are provided with teeth, firstly, to secure their food, also to make the first preparation of the food that nature's work may be properly performed.

"The stomach (so the organ to contain the food is named; if it is so treated that it becomes weak, all the other organs will grow weak) first receives the food immediately after it is prepared by being cut up by the teeth.

"Note the above, and take care not to cross the stomach too much, or to goad it, lest it become sensitive and in consequence inflict pain.

"However, the teeth are not exclusively preparers of the food. They have an important office as formers of the voice or speech. The tones of nature, and also of men, striking in proper order the teeth and the tongue, are so controlled that they become now the loud or again the charming voice.

"As Pliny says, 'The teeth are necessary to a goodly countenance and to speech.' There are some letters and words which cannot be properly spoken when the teeth have fallen out, or are worn and broken, or have grown short without being broken, and the voice then becomes harsh, without force, and unpleasant.

"When the teeth become bad it is natural that the mouth be kept shut to hide them.

"When a tooth is raised up and seems like a vegetable, to have grown large at the top, and makes one to suffer, it should be shown to an experienced physician with the request that he draw it out at once."

In the first chapter it states, in substance: Some, very few, however, are born with teeth. A few have instead of teeth a single bone taking their place. The teeth usually begin to grow the seventh month after birth, and keep on growing at intervals until there are thirty-two,—to some the teeth come easier, to others more painful; to some sooner, to others later. To many, as Pliny says, teeth grow in their eightieth year. To some, in old age, the teeth fall out and new ones grow, as happened to one man aged one hundred and four. He relates of another that he had two lower jaws, and on each several teeth. These latter are most likely some of Pliny's marvellous tales.

The second chapter, treating of the various causes of bad teeth, enjoins that whenever any mishap occurs to the teeth, which is often the case, it should be promptly met and counteracted by proper remedies before it becomes permanently settled. Care should be taken not only to see that no fragments of food remain between the teeth, but to wash and cleanse them with pure water. Indigestion should be avoided, as it produces vapors, rising from the stomach, which injure the teeth very materially. Vomiting also causes damage to the teeth, but as this is sometimes salutary, the mouth should be immediately refreshed and washed with rose-water and vinegar, or with any other purifier. All food detrimental to the teeth, such as dry figs, boiled honey, garlic, fat things, sour apples, and such things as set the teeth on edge, should be avoided. Care should be taken not to bite on hard things, to avoid too hot or over cold food; beware of quicksilver, or salves in which there is quicksilver, especially to take care if it is put on heated coals, its vapor does not enter the mouth and touch the teeth; in handling it, do not put the fingers in the mouth. Nor should we go to sleep after a heavy meal, for this will also damage the teeth. The precepts in this chapter are all credited to Avicenna and Mesue.

In the third chapter, how to assist children that their teeth may grow easily, it is advised, children to whom teething is painful should be often bathed, after which the gums should be gently rubbed with the finger, the finger having been first dipped in chicken-, goose-, or duck-fat, and pressed upon the spot where the teeth are supposed to make their eruption. It enjoins watchfulness, when the second teeth are erupting, that the first tooth is removed as soon as it is seen to be impeding the progress of the permanent tooth. If the second tooth has been pressed out of place as soon as the first tooth has been removed, the new tooth should be pushed every day towards the place where the first tooth was until it sits there and fits in among the others. This chapter is also credited to the writings of Avicenna and Mesue.

The fourth chapter, on toothache, begins by saying, "What toothache or pains in the teeth are, knows no one better than he who has experienced them, and I think there are no greater pains than these." Toothache may originate from a bad state or a destruction of the blood-vessels entering the teeth. Three ways of stopping the pain are given, credited to Johannes de Figo. They are, first, care in eating and drinking; second, by bleeding and purging; third, the taking of medicines, either simple or compound; these are of two kinds, one to stop the pain coming from heat, the other that coming from cold, a distinction, he adds, we cannot afford to lose sight of. Then follows a list of remedies it is not necessary to repeat. This chapter is credited to Johannes de Figo and Avicenna.

The fifth chapter, on hollow teeth, is particularly interesting. It treats of decay of the teeth, and, quoting from Mesue, says it may be stopped in three ways. First, by purging; second, by destroying the matter which hollows them out and eats them away. This is done by boiling cockel, that grows in rye and wheat, with vinegar, and holding it in the mouth, or vinegar in which capers-root with ginger is boiled; third, by getting rid of the hollow, which is done in two ways. The first is to scratch and clean with a fine chisel, knife, file, or with any other suitable instrument the parts attacked, and fill with gold leaves, for the preservation of the remaining part of the tooth. Presuming this to be reliable, the operation of filling cavities in carious teeth with gold, for the purpose of arresting this destructive process, was known to Mesue more than a thousand years ago.

The next chapter, on teeth on edge, contains nothing of interest. It is credited to Mesue and Figo.

Chapter VII., on yellow and black teeth, and the next on sleeping teeth, are of but little interest.

The ninth, on loose teeth, gives us an intimation of pyorrhœa. Teeth loosened by accident it directs to be bound to the sound teeth with silk or gold thread, using light and soft food and astringent mouth-washes.

The remaining chapters have no present interest.

Between this little book, compiled from authors most of whom had been dead when it was written from five hundred to more than a thousand years, and which may truly be said to belong to ancient dental literature, and the next which I show you, written by Bartholomew Eustachius in 1563, dental bibliography records but three works. Judging from the titles, I take them to be much like this which we have examined, and to possess but little other than historic interest. During this interval, however, modern anatomy was born, the chains of time-honored traditions were broken, the science of medicine and all that relates to it received a new impetus, and modern dental literature received its first contribution.

It requires courage, confidence, and indomitable energy to inaugurate a new departure that antagonizes to any marked extent the accepted doctrines of the day; far more did it require it when men were much less in a receptive mood than now, and were more firmly wedded to ideas that for a thousand years had been accepted without question. Andreas Vesalius was fully equal to the task when, in 1545 or thereabouts, he boldly disputed the anatomical teachings of Galen, dared to point out his errors, and contended that, as the greater part of Galen's descriptions had been made from monkeys, they did not correctly represent human anatomy. His audacity raised a crowd of vehement opponents, but although they silenced this doughty reformer, he was not vanquished; he did not quit the field until he saw the good work he had inaugurated fully established. He contended that anatomy must be studied from the cadaver itself; that the time-honored doctrines of long dead masters did not contain all there was to know. Having succeeded in awakening a spirit of inquiry, he retired from the professorship of anatomy at Padua he had so well filled, to accept a position at the court of Madrid, and was succeeded by Gabriel Fallopius, the discoverer of the Fallopian tube, a distinguished anatomist, who by his researches greatly advanced the science of special anatomy.

About this time Bartholomew Eustachius, the third master in anatomy of the triumvirate who are recognized as the fathers of modern anatomical science, the one with whom we as dentists have most to do, appeared upon the scene. His birthplace is unknown. He is said to have been a pupil of Vesalius at Padua, but he early settled at Rome, where he pursued his studies and practised as a physician. Although his genius was recognized, and he had wealthy patrons, he was very poor. He at times complained of his straightened circumstances, and that his meagre and uncertain income greatly hampered him in his work. An infirmity of temper, occasionally exhibited in his writings, and his absorbing devotion to anatomical studies, it has been suggested, may account for his poverty, and may also have prevented that full appreciation of his labors that did not come until long after his death. Although he was professor of anatomy at the College della Sapienze at Rome, he was but little known as a teacher, and his fame rests principally on his writings. His works are not numerous; indeed, even a long life is far too short to accomplish much of such exacting, painstaking, and careful work as that which Bartholomew Eustachius has presented to the world. The various papers which Eustachius produced during his life were collected together and published in a volume, entitled "Opuscula Anatomica," at Venice, 1563, this little volume which I show you, his work upon the teeth, once formed a part of that volume, it was torn from its companions when it reached some iconoclastic bookseller's hands. I have here the book complete, but of a much later date, published by Adrianum Beman, at Delphis, in 1726. Although published together in one volume, each treatise or essay was complete in itself. The first essay is upon the structure of the kidneys, to which is added much that has been done in that line by Vesalius. The second on the organ of hearing, in which for the first time is described the passage leading from the mouth to the ear, since known as the Eustachian tube. The third is on the bones, in which he enters into a controversy with Vesalius on the knowledge possessed by Galen of the structure of the human skeleton. Eustachius was inclined to defend Galen, or rather his teachings or doctrines, against the stricture of Vesalius. The last of these remarkable essays gives an account of the teeth, and was the first to enter fully into a history of their development. For this work he was fully equipped, his studies in embryology and comparative anatomy, so far in advance of any that had preceded him, were brought to bear upon this work, and have added much to its value. It is regarded as by far his best effort; indeed, in some respects he spoke as a prophet, describing conditions which he knew existed, but which were far too minute for human vision. Many years after his death the microscope revealed the accuracy of these deductions. It seems a pity that this work, so excellent, should have been so much neglected that to many well informed upon dental topics it is quite unknown.

In conclusion, permit me to present this work, "Tractatus de Dentibus," or a treatise upon the teeth, by Bartholomew Eustachius, as the first contribution to modern dental literature. It was not a chapter forming a part of some other work, but a work distinct and complete in itself. He says in the dedication "that he had collected together all he could find of that which had been written concerning the teeth; to this he added the results of his own labors in diligent original research, not in the effort to establish some preconceived doctrines or ideas, but rather in the spirit of an impartial and upright judge accustomed to place in order and sift evidence in the effort to reach the truth." His book is a systematic arrangement of so much of this data as, after careful study, proved worthy of acceptance. The title-page of the first edition, published under the supervision of the author, is particularly appropriate: "Bartholomaei Eustachii Sancto severinatis Libellus de Dentibus;" which, freely translated, may read, "Bartholomew Eustachius, his little book wholly devoted to the teeth."

It marks the dawn, as it were, of our specialties literature. The beginning of a long series of contributions giving the results of original investigations, of practical experiences, and of profound study, that has not only placed our profession fully abreast of others of the healing art, but in addition has added vastly to *their* resources and usefulness.

